# Growth Performance, Feed Utilisation, Endogenous Digestive Enzymes, Intestinal Morphology, and Antimicrobial Effect of Pacific White Shrimp (*Litopenaeus vannamei*) Fed with Feed Supplemented with Pineapple Waste Crude Extract as a Functional Feed Additive

**DOI:** 10.1155/2023/1160015

**Published:** 2023-04-01

**Authors:** Rungkan Klahan, Pinsurang Deevong, Jintana Wiboonsirikul, Bundit Yuangsoi

**Affiliations:** ^1^Faculty of Agricultural Technology, Phetchaburi Rajabhat University, Phetchaburi, Thailand; ^2^Faculty of Science, Kasetsart University, Bangkok, Thailand; ^3^Faculty of Agriculture, Khon Kaen University, Khon Kaen, Thailand

## Abstract

This study used pineapple waste crude extract (PWCE) to increase the potential of Pacific white shrimp (*Litopenaeus vannamei*) production for food sustainability and stability. The objective was to investigate the appropriate technique to increase the yield production and quality of shrimp and decrease waste from shrimp culture. Pacific white shrimp (average body size: 0.51 g) were fed with commercial feed supplemented with PWCE at various concentrations of 0 (control), 90, 170, and 250 ppt. Shrimp were fed five times per day for 80 days. At the end of the trial, the results showed that shrimp fed with the PWCE 250 ppt supplementation provided the highest growth rate and the best feed utilisation and yield (*P* < 0.05). The protein content of whole shrimp in all shrimp fed with the PWCE supplementation diet was higher than that in the control group (*P* < 0.05). On the contrary, the variation of endogenous digestive enzymes, including protease, trypsin, and the T/C ratio, was significantly lower in shrimp fed a diet supplemented with PWCE 250 ppt (*P* < 0.05). While in this group, the number of microorganisms on thiosulfate-citrate-bile salt-sucrose (TCBS), blood agar, and trypticase soy agar (TSA) was lowest (*P* < 0.05). Furthermore, the dietary PWCE at 250 ppt increased the volume of microvilli in the hindgut of shrimp, but the supplementation at 170 ppt improved the number of F-cells in the epithelial cells of the hepatopancreas. Nevertheless, the supplementation of PWCE in the diet did not affect the water quality (*P* > 0.05). Therefore, pineapple waste crude extract supplementation improves both quantitative and qualitative yields and tends to reduce waste.

## 1. Introduction

Pacific white shrimp is one of the economic aquaculture productions that has been adversely impacted by the coronavirus [1]. However, it was still produced in tremendous volume and provided the raising value and production by the data of global shrimp production higher in 2021 than in 2020 by at least 8.9%, while the growth of over 5% is forecasted for 2022 [2]. Nowadays, superintensive culture is used in shrimp production, which affects the high amount of feed and protein [3] and the amount of waste from feed residue and faeces. Feed is the main cost to produce aquatic animals for 30%–70% of the entire coast [4, 5]. Pacific white shrimp are carnivorous, requiring a high-protein feed ranging from 20% to 45% protein [4, 6]. The high protein level contained in the feed increases the price and is the main cost of production. In addition, the high-protein residue from the uneaten or undigested feed is a cause of waste in the form of nitrogen, which affects aquatic animal health [7]. The protein source in shrimp feed is mostly animal and plant by-products rather than fish meal, which is traditionally the main source of protein, due to the increasing cost of fish meal. Alternative protein ingredients may have a comparable protein level to high-quality fish meal (9FM) but might be less digestible and less easily absorbed, even though this feedstuff was modified before use to improve the availability and nutritional value of the raw materials [8]. Therefore, digested protein is the solution to this problem. The protein was digested with serine protease, which the hepatopancreas and microorganisms produce in the intestines of the shrimp [9]. Exogenous enzyme supplementation is the key alternative to the resolution of protein digestibility.

Functional feed additives are used to improve the animals' growth performance, immune response, physiological functions, and health performance compared to normal feed additives. Pineapple waste crude extract (PWCE) is the key functional feed additive alternative to solve this problem. Since the outstanding substance of PWCE is bromelain, it is well known that plant protease [10–12], the primary by-product from pineapple (*Ananas comosus* L. Merr.), represents between 50% and 65% of the total weight of the fruits and remains when peeled [13]. Fruit bromelain constitutes 30%–40% of the total fruit protein and represents almost 90% of the proteolytically active material of the pineapple fruit [10]. Bromelain belongs to a group of protein-digesting enzymes that attack the internal peptide bonds of the protein chain or endopeptidase [14]. Bromelain from pineapple waste was extracted from the peel, and the crown is a mixture of different thiol endopeptidases and other components, like phosphatase, glucosidase, peroxidase, cellulase, escharase, and several protease inhibitors [14–16]. Bromelain is a fusion of sulphur-containing enzymes and has had a broad range of therapeutic applications for almost six decades. It is widely utilised in animals to promote feed utilisation and health. Another essential property is its antimicrobial property.

Consequently, the substance is outstanding as an antimicrobial substance that presented the maximum zone of inhibition against all test pathogen saponin was extracted by dissolving it in ethanol [15, 17]. Furthermore, the activity of saponin and bromelain in *Ananas comosus* L. Merr. is membranolytic properties [18]. It contains other elements in smaller portions and is used as an anti-inflammatory agent, though scientists have also discovered its potential as an anticancer and antimicrobial agent. It has been reported as having positive effects on the respiratory, digestive, and circulatory systems and potentially on the immune system [16]. Moreover, the function of saponin was to promote nutrient absorption, digestive capacity, and growth performance in the shrimp's immune system and its resistance to pathogens [19].

Furthermore, the properties of saponin and bromelain on nutrient absorption promote digestive capacity and growth performance. The essential positive effect of saponins is promoting the shrimp's immune system and its resistance to pathogens [19]. In addition, bromelain is supposed to have antiviral, anti-inflammatory, and anticoagulatory activities that benefit shrimp health [20]. The properties of bromelain and saponin are more beneficial for shrimp health since the health problems have been caused by viruses, such as infectious hypodermal and hematopoietic necrosis virus (IHHNV), Taura syndrome viruses (TSV), and white spot syndrome virus (WSSV), and different species of bacteria, such as *Vibrio parahaemolyticus* [21]. In addition, white faeces syndrome (WFS) is a destructive disease for the global penaeid shrimp industry. WFS has been a critical threat to the shrimp industry since 2010 and was first reported in Thailand in 2009 [22]; the Southeast Asian countries, Thailand, Malaysia, Vietnam, Indonesia, China, and India in 2010 [22]; and Mexico in 2013 [23]. Nowadays, the causative agent of WFS remains unclear, but the primary cause was identified, such as a decrease in intestinal microbial diversity and an increase in the heterogeneity of intestinal microbiota [22]. Pathogen agent in the *Vibrio* genus seems to play an important role in WFS occurrence [24].

At present, the bromelain in PWCE is one protease used to supplement aquafeed. There are many types of bromelains used in aquafeed, such as those in crude extracts from *Rana rugulosa* [25], tilapia (*Oreochromis niloticus*) [26], grass carp (*Ctenopharyngodon idella*) [27], *Oreochromis mossambicus* [28], juvenile sterlet (*Acipenser ruthenus*) [29], and Pacific white shrimp [30]. Commercial bromelain powder is used as the feed additive in aquafeed for Pacific white shrimp [31]. The data indicated that bromelain was popular as a plant protease and functional feed additive in aquafeed, which promoted protein and feed utilisation and health. Therefore, this study is aimed at analyzing the possibility and optimal level of PWCE in promoting the quality and quantity of shrimp production, improving cost-effectiveness for the shrimp industry and safety concerns for consumers regarding antibiotics, and supporting pineapple farmers.

## 2. Materials and Methods

### 2.1. Ethical Statement

The procedures have been authorised by the Animal Ethics Committee for Experiments on Animals of Phetchaburi Rajabhat University and implemented in accordance with the experiment animal welfare regulations formulated by the Institute of Animals for Scientific Purposes Development (IAD) Thailand.

### 2.2. Shrimp Preparation

Ten thousand Pacific white shrimp (*Litopenaeus vannamei*) at postlarvae 12 (P12) were obtained from a private farm in Samut Sakhon, Thailand. They were acclimatised in a 1000 L fiber pond with 10 ppt saltwater and fed commercial pellet feed containing 40% protein five times a day for two weeks. The shrimp were randomly weighed throughout the experiment.

### 2.3. Enzyme Preparation and Specific Activity Assay

PWCE was collected from the crown and peel of pineapples (*Ananas comosus* L.) (“Batavia”) from a plantation in the Phetchaburi Province of Thailand. The pineapple at 1/3 dark green ripening stages fruit was used. The crown and peel were separated, cleaned, air-dried, weighed, and then chopped into small pieces. The samples were blended with cold distilled water at a 0.5 : 1 (*w*/*v*) ratio for 5 min using a multipurpose blender, and the blended liquid was filtered through a cloth sheet then centrifuged at 12,000 rpm at 4°C for 20 minutes. The supernatant (crude extract) was collected to determine the specific activity assay, following the procedure of Ketnawa et al. [32], and mixed with the feed.

### 2.4. Feed Trial Preparation

PWCE, at concentrations of 0, 90, 170, and 250 ml/1000 g feed, was added to a commercial sinking pellet feed containing 38% protein (Inteq®, Thailand) by adjusting the final volume of each trial to 250 ml. The pellets were coated with PWCE in liquid form by top spraying using a spray bottle and then dried at room temperature (30°C) for 1 h. After that, the pellets were coated with 1% fish oil (Prima, Ltd., Thailand) and then dried at room temperature for 24 h. The dry pellets were placed in a covered plastic box and stored at room temperature.

### 2.5. Experimental Procedure

The experiment was a completely randomised design (CRD) with four treatments randomly assigned to triplicate groups of shrimp. The initial weight of shrimp was 0.51 g and stocked into a 0.30 × 0.45 × 0.20 m glass tank at a density of 60 shrimp per cubic meter (20 individuals/glass tank). They were divided into 4 treatments (with 4 replications) based on the level of supplemented PWCE at 0, 90, 170, and 250 ppt shrimp fed five times a day to satiation for 80 days. During the experiment, the mortality and moulting were recorded daily, and the shrimp in each glass tank were counted and weighed monthly. The temperature and pH were measured daily, while the free ammonia, nitrite, alkalinity, calcium, and magnesium were detected weekly until the end of the experiment. The growth performance was monitored to determine the weight gain (WG), specific growth rate (SGR), average daily gain (ADG), survival rate (SR), feed intake (FI), feed conversion ratio (FCR), and protein efficiency ratio (PER), which were calculated according to the following equations by Hopkins [33]:

(1) $$\begin{array}{c} \text{WG}=\text{final weight }\left(\text{g}\right)–\text{initial weight }\left(\text{g}\right),\\ \text{SGR}=100\times \frac{\ln\text{ final weight }\left(\text{g}\right)–\ln\text{ initial weight }\left(\text{g}\right)}{\text{days}},\\ \text{ADG}=\text{final weight }\left(\text{g}\right)–\frac{\text{initial weight }\left(\text{g}\right)}{\text{experimental duration}},\\ \text{SR}=100\times \frac{\text{final number of fish}}{\text{initial number of fish}},\\ \text{FI}=\frac{\text{dry weight of feed}/\text{day of the experiment}}{\text{number of fish in each tank}},\\ \text{FCR}=\frac{\text{feed weight}}{\text{final weight }\left(\text{g}\right)–\text{initial weight }\left(\text{g}\right)},\\ \text{PER}=\frac{\text{wet weight gain }\left(\text{g}\right)}{\text{protein intake }\left(\text{g}\right)},\\ \text{ANPR}=\left[\frac{\text{Final body protein}-\text{initial body protein}}{\text{Apparent protein intake}}\right]\times 100. \end{array}$$

Water quality was detected every morning throughout the trial in terms of pH, temperature, and salinity using a 7-in-1 water quality meter (Yieryi®, China). Ammonia, nitrite, alkalinity, calcium, and magnesium were measured accordingly. At the end of the experiment, 5 shrimp from each replication (*n* = 20 shrimp/treatment) were collected from the hepatopancreas and hindgut to detect enzyme activity, pathology, and microorganisms, and the final whole body was analyzed for the proximate composition according to the AOAC method [34].

### 2.6. Chemical Composition

The whole body (with an exoskeleton) of white shrimp without hepatopancreas from each treatment about 20 shrimp was collected by random sampling at the end of the trial. The shrimp were dried at 105°C for 2 h in a hot air oven. The dried shrimp was ground and assayed for proximate analysis in terms of crude protein (Kjeldahl method), lipids (Soxhlet ether extract), ash, and crude fiber (Weende method).

### 2.7. Enzyme Activity

The hepatopancreas from white shrimp at the beginning of the trial (30 shrimp) and from each treatment (20 shrimp) at the end of the trial was collected to assay the activity of enzymes. Protease was assayed according to Gimenez et al. [35], while trypsin, chymotrypsin, and the T/C ratio were determined according to Rungruangsak-Torrissen et al. [36].

### 2.8. Antimicrobial Effect

The effect of PWCE on the number of pathogens and total bacteria in the hepatopancreas of white shrimp was investigated. The whole hepatopancreas was dissected from three shrimp of each treatment (supplemented with 90, 170, and 250 ppt of crude extract) and the control (without crude extract supplementation). The contents of each individual hepatopancreas were homogenized using a homogenizer pestle and serially diluted with normal saline (0.85% NaCl). The hepatopancreas suspension was spread in duplicate on two different media, thiosulfate-citrate-bile salt-sucrose agar (TCBS; Himedia™) and tryptic soy agar (TSA), for quantification of *Vibrio* species and total cultivable bacteria, respectively. Then, the TCBS and TSA agar plates were incubated at 28°C and 37°C, respectively. After incubation for 24 h, colony forming units (CFU) were counted, and the CFUs per gram of hepatopancreas content were calculated.

### 2.9. Hepatopancreatic Histology

At the terminal period, the hindgut and hepatopancreas of five shrimp in each tank were collected and fixed in a 10% neutral buffered formalin for histological analysis. The sections (4 *μ*m) were made using a rotary microtome and stained with haematoxylin and eosin (H&E) according to standard histology procedures. Tissue slides were digitally photographed with a light microscope (Nikon Eclipse Ci; ×10).

### 2.10. Statistical Analysis

Data obtained from the experiment except for pathology and antimicrobial effect, which had a completely randomised design with four replicates per treatment, were analyzed by one-way analysis of variance (ANOVA) followed by Duncan's multiple range tests. A significance level of *P* < 0.05 was used.

## 3. Results and Discussion

The growth performance in terms of final weight, WG, and SGR was the most significantly different in the shrimp fed with feed supplemented with PWCE at 250 ppt (*P* < 0.05). Moreover, the moulting and yield of this group were significantly higher than other groups (*P* < 0.05), while the ADG and SR were similar among groups (*P* > 0.05) (Table 1). The perfect growth rate of shrimp in the 250 ppt PWCE-supplemented group was according with the highest moulting rate of this group, and it caused the best growth rate because the growth rate of crustaceans depends on the frequency of moulting, which is related to many factors such as nutrients, water quality, and hormones. In the current study, nutrients, especially protein, had the biggest impact on moulting and growth rates. The protein utilisation and effective utilisation of feed were outstanding in this group. These results are in line with Arisa et al. [37] who found that the addition of both papain and bromelain enzymes in the feed (each at 1%) had a significant effect on the WG, daily growth rate, and SGR of *Litopenaeus vannamei*.

On the other hand, the relationship between the moulting cycle, which is the main factor in the growth of crustaceans, and the digestive enzyme is one of the important relationships to support the property of bromelain extracts from pineapple waste in stimulating the moulting and growth performance of white shrimp. The growth of crustaceans occurs by the shedding of the old exoskeleton and the formation of a new exoskeleton. The moult cycle in crustaceans is under the control of several regulatory hormones and internal and external factors [38], including rich nutrients like protein and lipids. The different stages of the moulting cycle in the red shrimp (*Pleoticus muelleri*) are related to proteolytic activity. Low trypsin and chymotrypsin activities were found during intermoult, and they increased during postmoult. A comparison of the protease-specific activity of the hepatopancreas of some species indicated a relationship between digestive enzyme activity and the feeding habits of the shrimp. However, omnivorous shrimp, such as *Litopenaeus vannamei* and *Penaeus monodon*, showed higher protease activity than carnivorous shrimp, *Penaeus californiensis* and *P. muelleri*. This indicated that the enzymatic activity in the hepatopancreas is related to feeding habits and the moulting cycle [39]. Furthermore, omnivorous shrimp, such as *P. vannamei* and *P. monodon*, showed higher protease activity in the hepatopancreas than *P. californiensis* and *P. muelleri* [40]. The omnivorous shrimp have higher protein requirements and protease activity in the hepatopancreas than the carnivorous species, and the proteolytic enzymatic activity in the hepatopancreas is influenced by the moulting cycle, feeding habits, and protein quality and quantity in feeds [39].

Furthermore, the study of Gimenez et al. [39] showed that the digestive proteinase activities in the hepatopancreas of *Artemesia longinaris* were highest during postmoult, and the trypsin and chymotrypsin activities were highest during intermoult. The effect of starvation after moulting in the white shrimp (*Penaeus vannamei*) was a decrease in the body weight and midgut weight because the protein accumulation in the body muscle is depressed to energy for living. The decrease in weight of the midgut is caused by the decrease in protein and water, which are the crucial substances in this tissue that effected on stimulants of the digestive system on digestive proteinase activities; however, fasting or inadequate feeding resulted in decreased proteolytic enzyme levels during the early premoult [41]. The relationship between moulting and enzymes was also found in crabs by Villasante et al. [42]. They determined that the digestive enzyme levels were different during the moulting cycle, including the colour and size of the hepatopancreas of *Callinectes* secaucus. Protease activity declines at the moulting stage and increases to an average level at the postmoult stage. In contrast, the lower level of protease at the premoult and moulting stages is affected by a decrease in feeding which causes the decline of amino acids in the muscle. Nevertheless, crab refeeding again more than usual in a postmoult stage affected the increase of protease level, amino acid content, and chitin synthesis for new exoskeleton synthesis. This factor affected the growth performance of crustaceans.

Therefore, supplementation with PWCE at the optimal level (250 ppt) could promote and, according to the high protein requirement, support the short-chain peptide and more accessible digestion protein, which is suitable with the low level of trypsin and chymotrypsin activities during the intermoult stage. Also, crustaceans in the premoult stage must accumulate nutrients, energy, and minerals by reabsorption from the old exoskeleton, which requires protease enzymes and other enzymes, such as chitinase. Thus, PWEC supplementation will support the lack of protease enzymes in this stage of the moulting cycle. In addition to using the properties of PWEC in white shrimp production, such as enhancing growth performance and feed utilisation in postlarvae Pacific white shrimp [30] and postlarvae *Litopenaeus vannamei* [37], there are also studies on the properties of bromelain crude extract in many kinds of fish, such as its effect on growth performance in Nile tilapia (*Oreochromis niloticus*) fingerlings and adults [26, 43]; innate immunity, disease resistance, and relative immune gene expression of Nile tilapia [44]; enhanced growth and immunity [45]; and improved growth performance, nonspecific humoral, and cell-mediated immunity of juvenile sterlet (*Acipenser ruthenus*) [29].

### 3.1. Feed Utilisation

The data regarding feed utilisation is shown in Table 2, in terms of feed intake and the feed conversation ratio, and protein utilisation, in terms of the protein efficiency ratio and ANPR, was the most promising in the shrimp fed with 250 ppt PWCE-supplemented feed (*P* < 0.05). According to the growth performance data, the shrimp in this group had the lowest feed cost and the highest benefit-cost ratio (B/C ratio) (*P* < 0.05).

The PWCE was an efficient action on protein utilisation presented in PER and ANPR because of the outstanding property or function of protein digestion by the bromelain substance. Bromelain belongs to a group of protein-digesting enzymes [46] which produce from the plant in terms of vegetable protease or plant protease. Bromelain from the PWCE is a mixture of different thiol endopeptidases and other components, like phosphatases, glucosidase, peroxidases, cellulases, glycoproteins, carbohydrates, and several protease inhibitors [15]. Bromelain has demonstrated positive effects on the digestive system and potentially on the immune system [16]. The trial's in vitro and in vivo studies demonstrate that bromelain exhibits various functions, like fibrinolytic, antioedematous, antithrombotic, and anti-inflammatory activities [46]. Bromelain has been successfully used as an exogenous digestive enzyme in various types of aquatic animals following a pancreatectomy in the case of a lack of exocrine pancreas or other intestinal disorders [15], including the high level of feed protein and difficulty digesting protein in feed. In addition, the factor that makes bromelain suitable for protein and feed utilisation enhancement is the wide pH range (4.5–9). Moreover, the substrate spectrum of the enzyme is broad, including synthetic low molecular mass amides and dipeptides up to high molecular weight substrates [15]. These reasons support the idea that bromelain has protease properties, and the bromelain from the crude extract promotes protein digestion and feed utilisation. The study of Sharma et al. [28] guarantees that bromelain has the substrate spectrum because this study used spirulina powder as the protein source in feed. Bromelain supplementation promotes in vitro protein digestibility which supports that bromelain-based supplementation could improve the digestibility of plant protein-based fish diets. In addition, the optimal pH for bromelain activity was still a wide range that was suitable and advantageous for protein digestibility. The optimal pH was 2.9–9, the optimum temperature was 37–60°C, and the molecular weight was 24.5–29 kDa [47–51].

It is well known that shrimp feed contains high amounts of protein, and nowadays, the protein source is made from a higher proportion of plant protein. Thus, exogenous proteases are the key to solving the problem presented in this study. This data is supported by the study of Bupphadong et al. [52] who reported that the concentrated pineapple extract increases in vitro digestibility of soybean meal protein which solves the problem regarding the digestibility of protein in feedstuff, leading to reduced undigested residues remaining in the digestive tract of shrimp. Many reports demonstrated that bromelain crude extract promotes protein and feed digestibility. The report of Klahan et al. [11] studied the digestibility of high-protein feed in shrimp and fish feed using commercial and crude-extracted bromelain and reported that both sources of bromelain were suitable to digest the protein in shrimp feed at pH 6 and 25–40°C. In addition, the current study was according to Klahan et al. [12], who found that the optimal condition for protein digestion with bromelain was pH 6 at 25°C for 5 and 30 min; the percentage of protein digestion was 63.15 and 70.66%. Moreover, they found the highest level of saponin content in the diet that was digested with crude bromelain extract at 170 and 250 ppt for 30 min (1.84 and 1.88 mg/g feed). This data determined that the bromelain from PWCE also contains saponin to support the digestibility of feed.

Saponin was found in PWCE because it is contained in pineapples. Saponin is a triterpenoid found in the plant and is nontoxic to crustaceans, such as shrimp and crab, but toxic to fish [17]. The study of Emmanuel and Deborah [53] reported that the amount of saponin contained in pineapple was 0.41 ± 0.03%. The subsistence or increase of saponin supported and promoted the growth rate, feed digestibility, and shrimp immunity. Because of this, the saponin and bromelain in the PWCE promoted nutrient absorption and helped to reduce waste caused by shrimp excretion [19]. The bromelain crude extract from pineapple waste could act on the complicated protein digestibility from plant protein in shrimp feed. In addition, the aqueous crude extract consisted of saponin, which was extracted by dissolving in ethanol [17]. Furthermore, both saponin and bromelain promote nutrient absorption, digestive capacity, and growth performance and provide positive effects in the shrimp's immune system and its resistance to pathogens [19]. Thus, both substances in the aqueous crude extract from pineapple by-products exhibited a positive effect on digestibility and shrimp production.

The results indicated that PWCE improved the protein digestion that affected the great PER and ANPR because the more accessible digested protein in feed, which predigested from PWCE to short-chain peptides and free amino acid, reduces the digestion energy and receives enough nutrients and energy. The energy and nutrients from the feed are more available for growth and other activities. Thus, enough nutrients and energy more than other groups affected lower feed intake in shrimp fed with 250 ppm of PWCE and also related to FCR and FCE that provided the crucial data in this group.

### 3.2. Water Quality

PWCE supplementation did not affect the water quality of the shrimp cultures, which were similar among groups (*P* > 0.05) (Table 3). The range of data for each parameter was within the water quality standards for Pacific white shrimp culture; however, the dissolved ammonia value trended slightly lower than other groups, which pointed to the improved relation with protein and feed utilisation of shrimp in this group.

The relationship between feed protein level and N waste excreta from shrimp depended on protein digestibility. From the results, nitrogen waste was not significantly different among groups in terms of total ammonia and nitrite. This relationship was described by Pongchawee [54], who studied nitrogen loading and water quality in closed prawn (*Macrobrachium rosenbergii* de Man, 1879) culture systems and determined that the duration after feeding was correlated with the ammonia gill excretion rate; prawns had the highest ammonia excretion rate after 1 hour of eating. Moreover, the soaking period was correlated with the rate of nitrogen loss from the feed. The nitrogen input from feed was converted to protein synthesis of prawns and organic nitrogen in prawn tissues, and the residues from feed intake would be wasted in the water. Thus, the most efficient digestibility of protein promoted a high level of protein utilisation and increased the rate of nitrogen conversion to organic nitrogen in shrimp tissue. Moreover, the highest protein digestibility still decreases nitrogen waste, which is also present in the uneaten feed. Although the value of dissolved nitrogen waste was similar among groups, the supplementation of PWCE tended to decrease the amount of nitrogen waste.

### 3.3. Flesh Quality with Proximate Composition

Flesh quality was analyzed from proximate analysis of the whole body with exoskeleton without hepatopancreas, which presented significant differences among groups (*P* < 0.05). Shrimp fed with PWCE-supplemented feed at 90–250 ppt showed a higher percentage of protein than the control group, and the percentage of ash was highest in shrimp fed with 250 ppt PWCE supplementation (*P* < 0.05); however, the percentage of fat was similar among groups (*P* > 0.05), while shrimp fed with 250 ppt PWCE-supplemented feed presented the lowest of the percentage of crude fiber (*P* < 0.05), as given in Table 4.

The percentage of protein in the flesh was highest in shrimp fed with PWCE supplementation because these groups have excellent protein digestibility. In addition, these groups received more accessible digestible protein and short-chain peptides from predigested protein by PWCE. For this reason, the energy for digestion and other nutrients, especially proteins and amino acids, was applied to nutrient accumulation in the muscle and other parts of the body. The main factors which affect the quality and quantity of shrimp productivity are feed quality, stocking density, and water quality [55–57]. The stocking density for both groups was 20 shrimp/tank, and the water quality did not differ, guaranteeing that the quality of feed from the supplementation of PWCE supported the protein deposition in the muscle. Protein turnover can be divided into its constituent processes—protein synthesis, protein growth, and protein degradation; protein growth is the net balance between protein synthesis and protein degradation. The high SGRs in invertebrates may be achieved by the relatively low rates of protein turnover and the relationships between diet composition, protein turnover, and amino acid metabolism in crustaceans. The dynamics of muscle protein deposition depend on the relationship between diet quality, protein synthesis, and muscle protein deposition. Furthermore, the energy level of the diet, protein digestibility, protein metabolism, amino acid requirements, and environmental factors, such as salinity, are the main factors affecting protein deposits in muscle [58]. According to the results of the current study, these reasons show that predigested protein in feed with PWCE supplementation promotes the small peptides that are products of proteolysis, which can be absorbed and utilised directly by the intestines [59]. Predigested shrimp feed with PWCE supplementation to produce semifeed hydrolysis has proved to be a potential protein source with a well-balanced amino acid profile [60, 61]. Also, the target of rapamycin (TOR) signaling pathway is a critical function that plays an essential role in balancing protein synthesis and degradation [62, 63]. The TOR signalling pathway has not been reported in *L. vannamei*; the study of Shao et al. [64] reported that TOR signaling pathway-related genes (tor, s6k, and 4e-bp) has improved expression levels in shrimp fed protein hydrolysates diet (PH), the dietary inclusion of fish meal (FM), and protein hydrolysate (PH) are presented tor and s6k expression levels of high regulated than control diet (CD). In addition, shrimp fed with FM and PH diets had significantly higher crude protein values of the muscle than those fed with the CD.

The crude fiber percentage in the shrimp's edible flesh decreased as PWCE increased (*P* < 0.05). The fiber in crustaceans comes from the exoskeleton and from the blood as chitin. Chitin, a structural polysaccharide built from chains of modified glucose, is the second most abundant natural polysaccharide, after cellulose, and is a linear polymer composed of repeating *β* (1,4)-N-acetylglucosamine units. Chitin exists in the exoskeletons of arthropods, such as crabs, shrimp, and insects, and is also produced by fungi and bacteria. Like cellulose, invertebrate animals can digest chitin on their own by breaking down the fibrous chitin into the glucose molecules that compose it. The structure of chitin affected the percentage of fiber in shrimp because the shrimp sample also included the exoskeleton. In addition, the level of chitin is related to moulting because the growth rate of crustaceans depends on moulting frequency and the younger shrimp moult more frequently than older shrimp. Thus, the results indicated that the percentage of fiber in shrimp fed with PWCE-supplemented feed at 250 ppt is close to the initial group and lower than in the other groups. Moreover, shrimp in this group have the highest moulting, which is related to the level of chitin as a fiber in shrimp. The percentage of ash presented the number of minerals in shrimp, which was highest in the initial group, followed by the shrimp fed with PWCE-supplemented feed at 250 ppt. The percentage of fiber which correlated with chitin and ash related to moulting supported the moulting data because the epicuticle is composed of only protein, whereas the exo- and endocuticle contain both chitin and protein in varying proportions depending on the type of cuticle, which is, whether it is rigid or flexible [65]. In addition, in many *Crustacea*, chitin is combined with calcium carbonate into laminae in a unique helical pattern [66, 67].

### 3.4. Endogenous Enzyme Activity

The variation of digestive enzyme activity in terms of protease, trypsin, chymotrypsin, and T/C ratio extracted from the hepatopancreas of Pacific white shrimp after being fed with a PWCE-supplemented diet for 80 days was significantly different among groups (*P* < 0.05), as shown in Table 5. The result pointed out that the activity of protease and trypsin of Pacific white shrimp fed with a 250 ppt PWCE-supplemented diet was lower than in other groups, especially the T/C ratio, which was the lowest (*P* < 0.05). The chymotrypsin activity was the lowest in the control group (*P* < 0.05).

The activity of the protease and protease group was trypsin, and chymotrypsin, including the T/C ratio, presented the lowest value in the 250 ppt PWCE-supplemented feed group, which is contrary to protein digestion and feed utilisation. The supplementation of PWCE, which is an exogenous enzyme, in the feed promoted the numerous short-chain peptides and free amino acids that do not need to be digested by an endogenous enzyme. This affects the reduction of enzyme production in shrimp. Furthermore, the activity of digestive enzymes is directly related to the digestion and absorption of nutrients [68]. Protease enzymes are the main digestive enzymes in the hepatopancreas of shrimp that digest proteins by breaking the peptide bonds, and they affect the digestion and absorption of nutrients [69]. The main types of proteases produced from the shrimp's hepatopancreas were serine proteases or the pancreatic enzyme group, such as trypsin and chymotrypsin, which are also activated in the hepatopancreas. They cleave proteins into free amino acids, which are easily absorbed by the cells of the hepatopancreas. The pancreas also produces a protein called pancreatic secretory trypsin inhibitor, which binds to trypsin and blocks its activity [70]. Thus, these reasons showed why the activity of all protease enzyme groups, especially trypsin, was the lowest. The results concluded that supplementation with PWCE at 250 ppt in feed provides short-chain proteins that are easier to digest, which decreases the activity of endogenous enzymes, and the free amino acids, which stimulate the secretion of trypsin inhibitor that affects the level of trypsin activity [71]. Figueiredo and Anderson [72] reported that the activity of the digestive enzymes in crustaceans was variable with feeding behaviour or habitat; various food integrity environments in different seasons affect the variation in enzyme activity in the digestive system as well.

In addition, the results were similar to the study of Luna-González et al. [73], who found that the activity of the protease enzyme, especially the serine protease, trypsin, and chymotrypsin, was reduced when the shrimp fed the feed decreased from 100% to only 80%–90% of the total feeding. This data confirmed that the shrimp fasting or fed with more accessible digested feed reduced the enzyme production, secretion, or action. Moreover, the residues of energy and nutrients were utilised in moulting and growth. Furthermore, these current results were in line with the trial of Nery et al. [74], who found that the gastrointestinal enzyme activity of white shrimp was inversely correlated with the weight of white shrimp fed only three times a day but raised in the biofloc system which had the best growth. In contrast, the enzyme activity was lower than in other groups.

### 3.5. Antimicrobial Effect

The crude extract from pineapple waste supplemented in feed affected the number of bacteria in the hepatopancreas of white shrimp. The CFUs of total bacteria and pathogenic bacteria per gram of hepatopancreas content are shown in Table 6.

The total numbers of cultivable bacteria on TSA of all treatments and the control (0 ppt or without crude extract supplementation) were not significantly different. The increase of bacterial number in the 90 ppt treatment was comparable to, but still higher than, the control and other treatments. This result suggested that the 90 ppt concentration of crude extract seems to be a better choice to enhance the growth potential of bacteria in the hepatopancreas with a 12.67-fold increase from the bacterial number of the control. However, a decreasing *Vibrio* colonial number was noticed on the TCBS agar. The *Vibrio* number was significantly reduced in the 250 ppt treatment by a 14.43-fold decrease, while the other treatments were not significantly different compared with the control. High concentrations of crude extract from pineapple waste (250 ppt) significantly inhibited the aquaculture pathogenic bacteria, *Vibrio* species, and did not significantly affect the total amount of bacteria in the hepatopancreas of the treated white shrimp.

### 3.6. Histological Analysis

**Figure 1:**
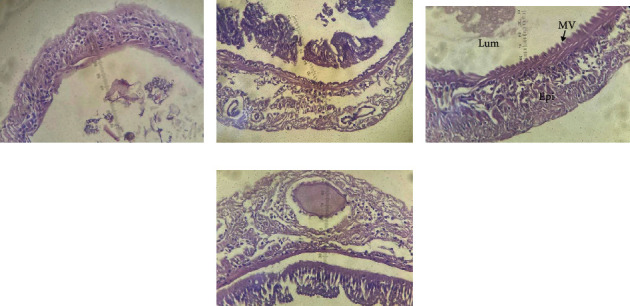
Light microscopic photograph of hindgut cross-section of Pacific white shrimp fed with pineapple waste crude extract supplementation at different levels for 80 days: (a) 0 ppt, (b) 90 ppt, (c) 170 ppt, and (d) 250 ppt (H&E, ×10). Epi = intestinal epithelium; Lum = intestinal lumen; MV = microvilli.

According to histological analysis, different levels of PWCE supplementation in diets affected the hindgut of *L. vannamei* (Figure 1). Overall, the intestinal structure of shrimp in all experimental groups was relatively intact with neat intestinal villi and smooth intestinal mucosa. The shrimp fed a diet supplemented with 170 ppt PWCE showed more hindgut villi than the other groups. This result indicated that the number of villi was likely to improve intestinal digestion and increase the absorption efficiency of nutrients.

**Figure 2:**
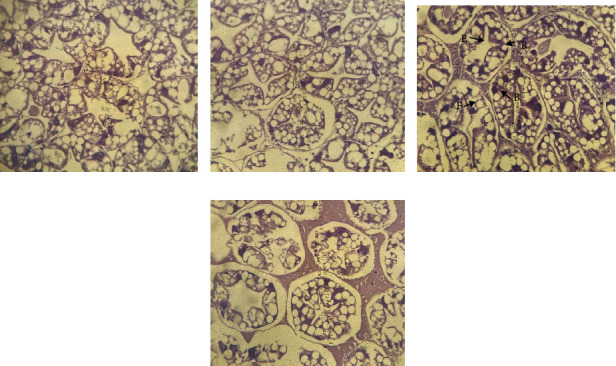
The hepatopancreatic in Pacific white shrimp fed with pineapple waste crude extract supplementation at different levels for 80 days: (a) 0 ppt, (b) 90 ppt, (c) 170 ppt, and (d) 250 ppt (H&E, ×10). E = embryonic; R = restorative; F = fibrillar; B = blister-like.

The shrimp hepatopancreas is composed of many hepatopancreas tubules. Four kinds of cells dominate the hepatopancreas tubules: E-cells (embryonic), R-cells (restorative), F-cells (fibrillar), and B-cells (blister-like). The shrimp fed with different levels of pineapple did not show a difference in all groups (Figure 2). On the other hand, the shrimp fed a diet supplemented with 170 ppt PWCE contained more F-cells in the hepatopancreas than the other treatments. F-cells are likely related to the synthesis of proteolytic enzymes and intracellular digestion, which aids in protein digestion and absorption. The hepatopancreas of white shrimp fed with 170 ppt PWCE-supplemented feed presented more F-cells than the shrimp fed with 250 ppt PWCE-supplemented feed, which is related with the high activity of protease, trypsin, and chymotrypsin. Because of this, the enzyme's activity is dependent on nutrient intake, especially protein related to the protease enzyme [75] reported that digestive enzymes in crustaceans might reflect different feeding habits and habitats. Thus, the 250 ppt PWCE-supplemented feed was more easily digested than the 170 ppt PWCE-supplemented feed, which affected the reduction of internal protease, resulting in numerous digestion and absorption cells being smaller as well [76]. The lower mRNA expression of digestive enzymes in shrimp fed with 80%–90% of the feeding ratio did not affect shrimp growth; this is the reason for better growth in the 250 ppt PWCE-supplemented feed group. In addition, the growth of crustaceans is due to moulting, which requires a large amount of energy; therefore, the shrimp do not require the energy to digest the feed [77]. Instead, this energy is used for moulting, resulting in faster, more frequent moulting [78]. In addition to obtaining sufficient nutrients, these groups of shrimp had better growth. In addition, B-cells may clear the hepatopancreas tubules from the rest of the digestion during the time between the absorption of nutrients and the secretion of new digestive enzymes [79].

## 4. Conclusions

The current study pointed out that pineapple waste crude extract (PWCE) can be a functional feed additive for promoting the moulting, feeding, protein utilisation, and growth performance of Pacific white shrimp (*Litopenaeus vannamei*). It was determined that the optimal level of supplementation was 250 ppt. Also, antimicrobial activity was found in this group, and histological analysis was performed.

## Figures and Tables

**Table 1:** Growth rate, survival rate, moulting, and yield of Pacific white shrimp fed with pineapple waste crude extract supplementation diet at different levels for 80 days.

Parameter	Pineapple waste crude extract supplementation (ppt)			
	0	90	170	250
Initial weight (g/shrimp)	0.51 ± 0.04^a^	0.51 ± 0.04^a^	0.51 ± 0.04^a^	0.51 ± 0.02^a^
Final weight (g/shrimp)	22.08 ± 2.09^b^	21.78 ± 0.67^b^	20.50 ± 1.03^b^	25.55 ± 0.26^a^
Weight gain (g/shrimp)	21.58 ± 2.02^b^	21.21 ± 0.70^b^	20.03 ± 1.00^b^	25.05 ± 0.26^a^
Average daily gain (g/shrimp/day)	0.29 ± 0.07^a^	0.29 ± 0.00^a^	0.26 ± 0.01^a^	0.32 ± 0.03^a^
Specific growth rate (%/day)	5.19 ± 0.06^ab^	5.08 ± 0.11^b^	5.15 ± 0.03^b^	5.38 ± 0.01^a^
Survival rate (%)	65.00 ± 7.07^a^	66.66 ± 2.88^a^	77.50 ± 10.60^a^	73.33 ± 5.77^a^
Moulting (moult)	51.33 ± 2.51^b^	51.00 ± 0.70^b^	57.50 ± 0.70^a^	57.00 ± 0.00^a^
Yield (g)	153.10 ± 6.54^b^	149.74 ± 0.13^b^	195.93 ± 3.35^ab^	203.31 ± 2.43^a^

Note: ^a,b^Means within a row with a different superscript are significantly different (*P* < 0.05).

**Table 2:** Feed utilisation and feed cost of Pacific white shrimp fed with pineapple waste crude extract supplementation diet at different levels for 80 days.

Parameter	Pineapple waste crude extract supplementation (ppt)			
	0	90	170	250
Feed intake (g/shrimp/day)	0.72 ± 0.13^b^	1.15 ± 0.14^a^	0.70 ± 0.13^b^	0.26 ± 0.03^c^
Feed conversion ratio	2.17 ± 0.43^b^	3.97 ± 0.61^a^	2.60 ± 0.36^b^	0.84 ± 0.04^c^
Protein efficiency ratio	1.23 ± 0.23^b^	0.60 ± 0.01^c^	1.01 ± 0.14^b^	3.13 ± 0.18^a^
Total feed intake (g/shrimp)	364.66 ± 53.25^b^	538.50 ± 79.90^a^	460.00 ± 14.14^ab^	162.66 ± 39.55^c^
Daily feed intake (%/day)	36.21 ± 2.08^c^	84.57 ± 10.11^a^	62.67 ± 1.28^b^	20.61 ± 2.35^d^
ANPR^1^ (%)	72.26 ± 2.04^b^	44.90 ± 1.14^b^	66.34 ± 3.31^b^	237.19 ± 19.08^a^
Feed coast (baht/kg)	88.00 ± 4.22^b^	123.99 ± 6.03^a^	97.17 ± 23.09^ab^	29.25 ± 2.43^c^
B/C ratio	1.59 ± 0.07^b^	1.13 ± 0.05^b^	1.48 ± 0.35^b^	4.80 ± 0.39^a^

Note: ^a,b,c^Means within a row with a different superscript are significantly different (*P* < 0.05). ^1^ANPR: apparent net protein retention.

**Table 3:** Water quality of Pacific white shrimp cultured by fed with pineapple waste crude extract supplementation diet at different levels for 80 days.

Water quality	Pineapple waste crude extract supplementation (ppt)			
	0	90	170	250
pH	7.67 ± 0.02^a^	7.62 ± 0.03^a^	7.62 ± 0.01^a^	7.64 ± 0.06^a^
Temperature (°C)	26.99 ± 0.44^a^	26.65 ± 0.37^a^	27.03 ± 0.51^a^	26.69 ± 0.13^a^
Salinity (ppt)	7.25 ± 0.18^a^	7.27 ± 0.12^a^	7.38 ± 0.08^a^	7.33 ± 0.03^a^
Ammonia (mg·L^−1^)	1.69 ± 0.45^a^	2.28 ± 0.26^a^	1.66 ± 0.87^a^	1.58 ± 0.63^a^
Nitrite (mg·L^−1^)	2.04 ± 0.34^a^	1.84 ± 0.61^a^	1.52 ± 0.90^a^	1.74 ± 0.52^a^
Alkalinity (mg·L^−1^)	128.81 ± 10.12^a^	127.56 ± 4.78^a^	128.16 ± 3.84^a^	137.76 ± 5.57^a^
Calcium (mg·L^−1^ as calcium carbonate)	314.66 ± 54.60^a^	316.66 ± 66.26^a^	288.00 ± 58.28^a^	320.91 ± 40.44^a^
Magnesium (mg·L^−1^ as calcium carbonate)	268.32 ± 36.85^a^	275.43 ± 26.00^a^	296.22 ± 27.09^a^	283.63 ± 49.89^a^

Note: ^a,b,c^Means within a row with a different superscript are significantly different (*P* < 0.05).

**Table 4:** Proximate composition (%) of Pacific white shrimp whole body without hepatopancreas (dry weight) fed with pineapple waste crude extract supplementation diet at different levels for 80 days.

Proximate composition	Initial	Pineapple waste crude extract supplementation (ppt)			
		0	90	170	250
Fat	2.44 ± 0.01^a^	2.35 ± 0.13^a^	2.41 ± 0.13^a^	2.37 ± 0.00^a^	2.44 ± 0.02^a^
Crude protein	69.66 ± 0.50^c^	73.72 ± 0.92^b^	77.34 ± 0.23^a^	76.06 ± 0.80^a^	76.51 ± 0.50^a^
Crude fiber	5.42 ± 0.02^c^	6.30 ± 0.11^a^	6.17 ± 0.04^a^	6.06 ± 0.35^ab^	5.84 ± 0.07^b^
Ash	12.17 ± 0.02^a^	10.62 ± 0.20^c^	10.68 ± 0.06^c^	10.09 ± 0.58^c^	11.56 ± 0.14^b^

Note: ^a,b,c^Means within a row with a different superscript are significantly different (*P* < 0.05).

**Table 5:** Digestive enzyme activity (U/mg protein/min) from hepatopancreas of Pacific white shrimp fed with pineapple waste crude extract supplementation diet at different levels for 80 days.

Enzyme	Initial	Pineapple waste crude extract supplementation (ppt)			
		0	90	170	250
Protease	2.11 ± 0.00^a^	0.57 ± 0.00^c^	0.58 ± 0.01^c^	0.64 ± 0.02^b^	0.50 ± 0.01^d^
Trypsin	457.72 ± 4.99^a^	48.38 ± 0.97^d^	100.15 ± 9.07^b^	80.04 ± 3.20^c^	53.61 ± 6.68^d^
Chymotrypsin	238.94 ± 2.99^a^	33.76 ± 1.70^d^	55.96 ± 0.80^c^	59.43 ± 0.35^b^	53.04 ± 0.78^c^
T/C ratio	1.91 ± 0.00^a^	1.44 ± 0.10^b^	1.78 ± 0.13^a^	1.34 ± 0.04^b^	1.01 ± 0.14^c^

Note: ^a,b,c^Means within a row with a different superscript are significantly different (*P* < 0.05).

**Table 6:** Colony forming unit (CFU/g) of cultivable bacteria in hepatopancreas content of Pacific white shrimp fed with pineapple waste crude extract supplementation diet at different levels for 80 days.

Cultivable bacteria on agar medium	Pineapple waste crude extract supplementation (ppt)			
	0	90	170	250
*Vibrio* sp. on TCBS	6.11 ± 1.23 × 10^5^^a^	4.66 ± 0.07 × 10^5^^a^	5.10 ± 0.03 × 10^5^^a^	3.96 ± 0.70 × 10^4^^b^
Total bacteria on TSA	1.80 ± 0.58 × 10^6^	2.46 ± 1.58 × 10^7^^c^	3.60 ± 0.52 × 10^6^^c^	5.40 ± 2.06 × 10^5c^

Data are the mean ± STDEV (*n* = 3); cfu: colony forming unit. Different superscripts in the same row indicate significant difference (*P* < 0.05).

## Data Availability

All data generated or analyzed during this study are included in this article.

## References

[1] Howell M. How has India’s shrimp sector weathered the Covid-19 crisis?. https://thefishsite.com/articles/how-has-indias-shrimp-sector-weathered-the-covid-19-crisis.

[2] Fletcher R. Global shrimp production sees significant growth in 2021. https://thefishsite.com/articles/global-shrimp-production-sees-significant-growth-in-2021-gorjan-nikolik-rabobank.

[3] Taukhid I., Tampangallo B. R., Asaad A. I. J. (2021). Application of sludge collector in super-intensive vannamei shrimp farms. *IOP Conference Series: Earth and Environmental Science*.

[4] Halver J. E., Hardy R. W. (2002). *Fish Nutrition*.

[5] Das A. P. U., Tanmoy G. C., Mandal S. C. (2012). Compensatory growth in fishes a boon to aquaculture. *Aquaculture Europe*.

[6] Lee C., Lee K. Y. (2018). Dietary protein requirement of Pacific white shrimp *Litopenaeus vannamei* in three different growth stages. *Fisheries and Aquatic Sciences*.

[7] Lazzari R., Baldisserotto B. (2008). Nitrogen and phosphorus waste in fish farming. *Boletim do Instituto de Pesca*.

[8] Wangsoontorn S., Chuchird N., Wudtisin I., Crook A. (2018). The effect of substituting fish meal with fermented soybean meal on the growth performance and immune parameters of Pacific white shrimp (*Litopenaeus vannamei*). *Journal of Fisheries and Environment*.

[9] Senphan T., Benjakul S., Kishimura H. (2015). Purification and characterization of trypsin from hepatopancreas of Pacific white shrimp. *Journal of Food Biochemistry*.

[10] Rowan A. D. (2013). Fruit bromelain. *Handbook of proteolytic enzyme*.

[11] Klahan R., Youngsoi B., Wiboonsirikul J., Deewong P. (2021). The evaluation of protein digestibility, saponin and trypsin inhibitor content in pacific white shrimp (*Litopenaeus vanamei*) feed, digested with bromelain crude extract from pineapple waste. *Proceedings of the International Conference on Fisheries and Aquaculture*.

[12] Klahan R., Wiboonsirikul J., Yuangsoi B. The comparison of *in vitro* protein digestibility in high protein aquafeed with commercial bromelain and bromelain crude extracted from peel and crown of pineapple at various temperature and pH.

[13] Gunwantrao B. B., Bhausaheb S. K., Ramrao B. S., Subhash K. S. (2016). Antimicrobial activity and phytochemical analysis of orange (*Citrus aurantium* L.) and pineapple (*Ananas comosus* (L.) Merr.) peel extract. *Annals of Phytomedicine*.

[14] Wijeratnam S. W., Finglas P. M., Toldrá F. (2016). Pineapple. *Encyclopedia of Food and Health*.

[15] Bhattacharyya B. K. (2008). Bromelain : an overview. *Natural Product Radiance*.

[16] Chakraborty A. J., Mitra S., Tallei T. E. (2021). Bromelain a potential bioactive compound: a comprehensive overview from a pharmacological perspective. *Life*.

[17] Amalia M., Alvionita M., Susanti S. E., Dian N. (2020). Isolation, identification, and inhibition of saponin isolates from pineapple (*Ananas comosus* L.) and candlenut (*Aleurites moluccanus* L.) against xanthine oxidase by *in vitro* assay. *IOP Conference Series: Materials Science and Engineering*.

[18] Zharfan R. S., Purwono P. B., Mustika A. (2017). Antimicrobial activity of pineapple (*Ananas comosus* L. Merr) extract against multidrug-resistant of Pseudomonas aeruginosa: an in vitro study. *Indonesian Journal of Tropical and Infectious Disease*.

[19] Acosta R., Rosen Y., Ariav R. The use of saponins in aquaculture. https://aquafeed.co.uk/the-use-of-saponins-in-aquaculture-21026.

[20] Hikisz P., Bernasinska S. J. (2021). Beneficial properties of bromelain. *Nutrients*.

[21] Alavandi S. V., Muralidhar M., Syama D. J. (2019). Investigation on the infectious nature of Running Mortality Syndrome (RMS) of farmed Pacific white leg shrimp, *Penaeus vannamei* in shrimp farms of India. *Aquaculture*.

[22] Tamilarasu A., Nethaji M., Bharathi S., Chrispin C. L., Lingam R. S. S. (2020). Review on the emerging white feces syndrome in shrimp industry. *Journal of Entomology and Zoology Studies*.

[23] López-Téllez N. A., Corbalá-Bermejo J. A., Bustamante-Unzueta M. L., Silva-Ledesma L. P., Vidal-Martínez V. M., Rodriguez-Canul R. (2020). History, impact, and status of infectious diseases of the Pacific white shrimp *Penaeus vannamei* (Bonne, 1831) cultivated in Mexico. *Journal of the World Aquaculture Society*.

[24] Somboon M., Purivirojkul W., Limsuwan C., Chuchird N. (2012). Effect of Vibrio spp. in white feces infected shrimp in Chanthaburi, Thailand. *Fisheries Research Bulletin*.

[25] Klahan R., Sirithanawong B. (2015). Growth performance and feed utilization of common lowland frog (*Rana rugulosa* Wiegmann) fed with supplementation by bromelain extracted from pineapple feed. *International Journal of Engineering Research and Development*.

[26] Yuangsoi B., Klahan R., Charoenwattanasak S., Lin S. M. (2018). Effects of supplementation of pineapple waste extract in diet of Nile tilapia (*Oreochromis niloticus*) on growth, feed utilization, and nitrogen excretion. *Journal of Applied Aquaculture*.

[27] Choi W. M., Lam C. L., Mo W. Y., Wong M. H. (2016). Upgrading food wastes by means of bromelain and papain to enhance growth and immunity of grass carp (*Ctenopharyngodon idella*). *Environmental Science and Pollution Research*.

[28] Sharma S. A., Surveswaran S., Arulraj J., Velayudhannair K. (2021). Bromelain enhances digestibility of spirulina-based fish feed. *Journal of Applied Phycology*.

[29] Wiszniewski G., Jarmołowicz S., Hassaan M. S. (2019). The use of bromelain as a feed additive in fish diets: growth performance, intestinal morphology, digestive enzyme and immune response of juvenile Sterlet (*Acipenser ruthenus*). *Aquaculture Nutrition*.

[30] Klahan R., Onsuwan P., Limprachya S. Growth performance and feed utilization of white leg shrimp fed with crude extract from crown and peel of pineapple supplemented feed.

[31] Klahan R., Maliyaem P., Pungneat R. Growth performance, feed utilization and water quality of Pacific white shrimp fed with bromelain powder supplementation diet.

[32] Ketnawa S., Chaiwut P., Rawdkuen S. (2012). Pineapple wastes: a potential source for bromelain extraction. *Food and Bioproducts Processing*.

[33] Hopkins K. D. (1992). Reporting fish growth: a review of the basics1. *Journal of the World Aquaculture Society*.

[34] A.O.A.C. (2000). *Official Methods of Analysis*.

[35] Gimenez M. I., Studdert C. A., Sanchez J. J, De Castro R. E. (2000). Extracellular protease of *Natrialba magadii*: Purification and biochemical characterization. *Extremophiles*.

[36] Rungruangsak-Torrissen K., Sunde J., Berg A. E., Nordgarden U., Fjelldal P. G., Oppedal F. (2009). Digestive efficiency, free amino acid pools and quality of growth performance in Atlantic salmon (*Salmo salar* L.) affected by light regimes and vaccine types. *Fish Physiology and Biochemistry*.

[37] Arisa I. I., Muchlisin Z. A., Purba S., Muhammadar A. A., Mellisa S. (2021). The effect of papain and bromelain enzymes on the growth and feed utilization of post larvae *Litopenaeus vannamei*. *IOP Conference Series: Earth and Environmental Science*.

[38] Hosamani N., Srinivasa R. B., Ramachandra R. P. (2017). Crustacean molting: regulation and effects of environmental toxicants. *Journal of Marine Science: Research and Development*.

[39] Gimenez A. V. F., Garcıa-Carreno F. L., Navarrete del Toro M. A., Fenucci J. L. (2002). Digestive proteinases of *Artemesia longinaris* (Decapoda, Penaeidae) and relationship with molting. *Comparative Biochemistry and Physiology Part B*.

[40] Fernández Gimenez A. V., García-Carreño F. L., Navarrete del Toro M. A., Fenucci J. L. (2001). Digestive proteinases of red shrimp *Pleoticus muelleri* (Decapoda, Penaeoidea): partial characterization and relationship with molting. *Comparative Biochemistry and Physiology Part B: Biochemistry and Molecular Biology*.

[41] Muhlia-Almazán A., García-Carreño F. L. (2002). Influence of molting and starvation on the synthesis of proteolytic enzymes in the midgut gland of the white shrimp _Penaeus_ _vannamei_. *Comparative Biochemistry and Physiology Part B: Biochemistry and Molecular Biology*.

[42] Villasante V. F., Fernandez I., Preciado R. M., Oliva M., Tova D., Nolasco H. (1999). The activity of digestive enzyme during the molting stage of the arched swimming Callinectes arcautus Orday. 1863. (Crustacea : Decapoda : Portunidae). *Bulletin of Marine Science*.

[43] Sukri S. A. M., Zuharlida Y. A., Shazani T. H. (2022). Effect of feeding pineapple waste on growth performance, texture quality and flesh colour of nile tilapia (*Oreochromis niloticus*) fingerlings. *Saudi Journal of Biological Sciences*.

[44] Doan H. V., Hoseinifar S. H., Harikrishnan R. (2021). Impacts of pineapple peel powder on growth performance, innate immunity, disease resistance, and relative immune gene expression of Nile tilapia, *Oreochromis niloticus*. *Fish & Shellfish Immunology*.

[45] Mo W. Y., Choi W. M., Man K. Y., Wong M. H. (2020). Food waste-based pellets for feeding grass carp (*Ctenopharyngodon idellus*): Adding baker's yeast and enzymes to enhance growth and immunity. *Science of the Total Environment*.

[46] Pavan R., Jain S., Shraddha K. A. (2012). Properties and therapeutic application of bromelain: a review. *Biotechnology Research International*.

[47] Lopes F. L. G., Júnior J. B. S., de Souza R. R., Ehrhardt D. D., Santana J. C. C., Tambourgi E. B. (2009). Concentration by membrane separation processes of a medicinal product obtained from pineapple pulp. *Brazilian Archives of Biology and Technology*.

[48] Corzo C. A., Waliszewski K. N., Welti-Chanes J. (2012). Pineapple fruit bromelain affinity to different protein substrates. *Food Chemistry*.

[49] Ketnawa S., Chaiwut P., Rawdkuen S. (2011). Aqueous two-phase extraction of bromelain from pineapple peels (‘Phu Lae’ cultv.) and its biochemical properties. *Food Science and Biotechnology*.

[50] Ketnawa S., Chaiwut P., Rawdkuen S. (2011). Extraction of bromelain from pineapple peels. *Food Science and Technology International*.

[51] Bala M., Amid A., Mel M., Jami M. S., Salleh H. M. (2012). Recovery of recombinant bromelain from Escherichia coli BL21-AI. *African Journal of Biotechnology*.

[52] Bupphadong F., Chirapongsatonkul N., Kuepethkaew S., Klomklao S., U-taynapun K. (2019). Concentrated pineapple extract and Na-Cid® facilitates the digestion of soybean meal and shrimp feed. *International Journal of Agricultural Technology*.

[53] Emmanuel E., Deborah S. (2018). Phytochemical and anti-nutritional studies on some commonly consumed fruits in Lokoja, Kogi state of Nigeria. *General Medicine Open*.

[54] Pongchawee K. (2018). Nitrogen loading and water quality in closed prawn (*Macrobrachium rosenbergii* de Man, 1879) culture systems. *Electronic Fisheries Journal*.

[55] Hopkins J. S., Hamilton R. D., Sandier P. A., Browdy C. L., Stokes A. D. (1993). Effect of water exchange rate on production, water quality, effluent characteristics and nitrogen budgets of intensive shrimp ponds. *Journal of the World Aquaculture Society*.

[56] Gaber M. M., Omar E. A., Abdel-Rahim M., Nour A. M., Zaki M. A., Srour T. M. (2012). Effects of stocking density and water exchange rates on growth performance of tiger shrimp, Penaeus semisulcatus cultured in earthen ponds. *Journal of Aquaculture Research & Development*.

[57] Cruz-Suárez L. E., Ricque-Marie D., Martínez-Vega J. A., Wesche-Ebeling P. (1993). Evaluation of two shrimp by-product meals as protein sources in diets for *Penaeus vannamei*. *Aquaculture*.

[58] Mente E. (2006). Protein nutrition in *crustaceans*. *Resources*.

[59] Gilbert E. R., Wong E. A., Webb K. E. (2008). Board-invited review: peptide absorption and utilization: implications for animal nutrition and health. *Journal of Animal Science*.

[60] Chalamaiah M., Kumar B. D., Hemalatha R., Jyothirmayi T. (2012). Fish protein hydrolysates: proximate composition, amino acid composition, antioxidant activities and applications: a review. *Food Chemistry*.

[61] Cai L., Wu X., Zhang Y., Li X., Ma S., Li J. (2015). Purification and characterization of three antioxidant peptides from protein hydrolysate of grass carp (*Ctenopharyngodon idella*) skin. *Journal of Functional Foods*.

[62] Wullschleger S., Loewith R., Hall M. N. (2006). TOR signaling in growth and metabolism. *Cell*.

[63] Sun X., Wheeler C. T., Yolitz J. (2014). A Mitochondrial ATP Synthase Subunit Interacts with TOR Signaling to Modulate Protein Homeostasis and Lifespan in *Drosophila*. *Cell Reports*.

[64] Shao J., Zhao W., Liu X., Wang L. (2018). Growth performance, digestive enzymes, and TOR signaling pathway of *Litopenaeus vannamei* are not significantly affected by dietary protein hydrolysates in practical conditions. *Frontiers in Physiology*.

[65] Riddiford L. M., Resh V. H., Cardé R. T. (2009). Molting. *Encyclopedia of Insects*.

[66] Cheng L., Wang L., Karlsson A. M. (2008). Image analyses of two crustacean exoskeletons and implications of the exoskeletal microstructure on the mechanical behavior. *Journal of Materials Research*.

[67] Astrop T. I., Sahni V., Blackledge T. A., Stark A. Y. (2015). Mechanical properties of the chitin-calcium-phosphate “clam shrimp” carapace (Branchiopoda: Spinicaudata): implications for taphonomy and fossilization. *Journal of Crustacean Biology*.

[68] Xia S., Zhao W., Li M. (2018). Effects of dietary protein levels on the activity of the digestive enzyme of albino and normal *Apostichopus japonicus* (Selenka). *Aquaculture Research*.

[69] Muhlia-Almazán A., Garcıa-Carreno F. L., Sanchez-Paz J. A., Yepiz-Plascencia G., Peregrino-Uriarte A. B. (2003). Effects of dietary protein on the activity and mRNA level of trypsin in the midgut gland of the white shrimp *Penaeus vannamei*. *Comparative Biochemistry and Physiology Part B: Biochemistry and Molecular Biology*.

[70] Wang G.-P., Xu C.-S. (2010). Pancreatic secretory trypsin inhibitor: more than a trypsin inhibitor. *World Journal of Gastrointestinal Pathophysiology*.

[71] Williams J. A., Johnson L. R. (2004). Synthesis and activation of trypsin. *Encyclopedia of Gastroenterology*.

[72] Figueiredo M. S. R. B., Anderson A. J. (2009). Digestive enzyme spectra in crustacean decapods (Paleomonidae, Portunidae and Penaeidae) feeding in the natural habitat. *Aquaculture Nutrition*.

[73] Luna-González A., Ávila-Leal J., Fierro-Coronado J. A. (2017). Effects of bacilli, molasses, and reducing feeding rate on biofloc formation, growth, and gene expression in *Litopenaeus vannamei* cultured with zero water exchange. *Latin American Journal of Aquatic Research*.

[74] Nery R. C., Costa C. B., Rodrigues F., Soares R., de Souza Bezerra R., Peixoto S. (2019). Effect of feeding frequency on growth and digestive enzyme activity in *Litopenaeus vannamei* during the grow-out phase in biofloc system. *Aquaculture Nutrition*.

[75] Johnston D., Freema J. (2005). Dietary preference and digestive enzyme activities as indicators of trophic resource utilization by six species of crab. *The Biological Bulletin*.

[76] Córdova-Murueta J. H., Navarrete-del-Toro M. A., García-Carreño F. L. (2017). Advances in the study of activity additivity of supplemented proteases to improve digestion of feed protein by *Penaeus vannamei*. *Aquaculture Nutrition*.

[77] Yuan Y., Jin M., Fang F. (2022). New insight into the molting and growth in crustaceans: regulation of energy homeostasis through the lipid *nutrition*. *Science*.

[78] Saravanan S., Kamalam B. S., Kumar J. S. S. (2008). Moulting and behaviour changes in freshwater prawn. *Shellfish News*.

[79] Vogt G. (1993). Differentiation of B-cells in the hepatopancreas of the Prawn *Penaeus monodon*. *Acta Zoologica*.

